# Recent Developments in Industrial Mycozymes: A Current Appraisal

**DOI:** 10.1080/21501203.2021.1974111

**Published:** 2021-09-16

**Authors:** Suresh Nath, Naveen Kango

**Affiliations:** Department of Microbiology, Dr. Harisingh Gour Vishwavidyalaya, Sagar, MP, India

**Keywords:** Fungi, Enzyme, Mycozyme, Cellulase, Amylase

## Abstract

Fungi, being natural decomposers, are the most potent, ubiquitous and versatile sources of industrial enzymes. About 60% of market share of industrial enzymes is sourced from filamentous fungi and yeasts. Mycozymes (*myco-fungus; zymes-enzymes*) are playing a pivotal role in several industrial applications and a number of potential applications are in the offing. The field of mycozyme production, while maintaining the old traditional methods, has also witnessed a sea change due to advents in recombinant DNA technology, optimisation protocols, fermentation technology and systems biology. Consolidated bioprocessing of abundant lignocellulosic biomass and complex polysaccharides is being explored at an unprecedented pace and a number of mycozymes of diverse fungal origins are being explored using suitable platforms. The present review attempts to revisit the current status of various mycozymes, screening and production strategies and applications thereof.

## Introduction

Modern biologists classify fungi as lower eukaryotes as they show absorptive mode of nutrition. Fungi being obligate heterotrophs secrete an array of extracellular mycozymes to hydrolyse complex polymeric substrates around. Many times, these mycozymes are resilient enough to survive harsh conditions including acidic pH, low water activity level and high temperature. Solid state fermentation (SSF) of complex substrates, particularly agro-industrial wastes such as sugarcane bagasse, palm kernel cake, copra meal, wheat bran, etc. is naturally suitable for the fact that molds thrive well in xerophilic conditions.

The first use of the mycozymes goes back to the beginning of the 19^th^ century. The first mycozyme, diastase, was known in the year 1833 (Payen and Persoz 1833; Asimov 1982) and the term enzyme was first used in 1877, which meant “*in leaven*” in Greek (Kuhne 1877). The first reference to the successful application of mycozymes, however, was taka-diastase from *Aspergillus oryzae* in the year 1894 to produce koji cultivated on rice by Jokichi Takamine. In 1897, yeast extract, free of yeast cells was used for sucrose hydrolysis. J.M. Nelson and E.G. Griffin, in 1916, demonstrated the immobilisation of invertase from yeast on charcoal and alumina and showed that enzymes can be reused (Bennett and Frieden 1969). Since then, a number of enzymes of fungal origin have been discovered and are exploited commercially in various industries. Most enzymes used in industries belong to the hydrolase class, i.e. enzymes that degrade various complex polymeric natural substances e.g. cellulose, starch, xylan and protein. The advancement in the fermentation processes lead to the large-scale manufacturing of mycozymes. Post 1980, mycozyme production was further improved by the application of genetic engineering, heterologous expression and efforts were made to alter characteristics of the fungal enzymes by protein engineering to acquire desirable properties suitable for industrial applications.

Selection of commercially important strain for mycozyme production is a very significant step in ascertaining economic feasibility of the enzyme production and application. This further helps in selecting the substrate, reaction conditions and recovery methods, which are economically favourable. The global enzyme market size was estimated at USD 9.9 billion in 2019 and is anticipated to grow at a compound annual growth rate (CAGR) of 7.1% from 2020 to 2027 and among all, more than 50% enzymes will be of fungal origin (https://www.grandviewresearch.com/industry-analysis/enzymes-industry). Increasing demand from user industries such as food, beverage, biofuel, animal feed, and home cleaning, is expected to drive the market growth over the forecast period. Increasing health awareness among consumers has resulted in the growing consumption of functional food products, which is expected to propel the product demand soon. Role of mycozymes in generation of health promoting prebiotic oligosaccharides and nutraceuticals has been the recent spurt of interest (Jana et al. 2018; Choukade and Kango 2019; Khangwal et al. 2020).

Apart from the conventional approaches, mycozymes are being explored using modern tools such as gene cloning and editing, directed evolution, protein engineering, molecular dynamic simulation etc. As a result, interesting chimeric enzymes, overexpression systems and robust enzymes are being produced and their functional patterns are being unravelled. Recently, Gilmore et al. (2020) reported preservation of the protein domain order from *Piromyces finnis* wherein, the chimeric enzymes retained catalytic activity at temperatures over 80°C and were able to associate with cellulosomes purified from this anaerobic fungus.

In this review, we explore the current status of mycozymes, and the mechanism involved in their extracellular secretion, their UpToDate relevance in different industries and the advents made in their production and applications. The recent advancements in genetic engineering of these industrial enzymes and its impact on the enzyme due to gene cloning and expression in different hosts are also being explored in this review.

## 1. Regulatory aspects of mycozyme secretion

**Figure 1.**
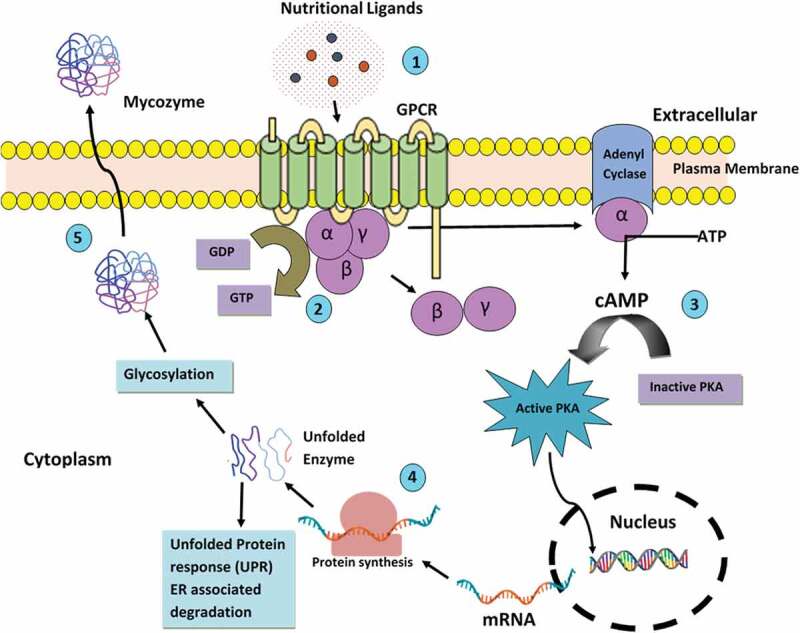
Regulatory aspects of mycozyme expression: Schematic illustration of G-protein signalling (1) Nutritional ligands bind to G protein-coupled receptor (GPCR) and (2) trigger the GDP-GTP exchange on the Gα protein coupled with dissociation of Gα and Gβγ (3) Gα triggers downstream signal cascades, which include the Cyclic AMP (cAMP) activated Protein Kinase A (PKA) pathway (4) Enzyme synthesis, post-translational folding and glycosylation and (5) extracellular secretion (Peberdy 1994; Baker 2018).

Filamentous fungi are a well-known source of industrial enzymes because of their remarkable capability for their extracellular production (Jun et al. 2011). Extracellular enzymes are synthesised inside the fungal cell and are then secreted in the extracellular environment, where their function is to catalyse transformation of complex substrates into smaller molecules which would be later taken up by fungus for its growth and development. Their potential to secrete hydrolases for digesting most complex macromolecules has been exploited for various applications at large-scale (Table 1). Baker (2018) hypothesised three different biological processes that contribute to secretion of inducible mycozymes by filamentous fungi, (1) nutrient sensing, (2) transcriptional regulation and (3) translation and secretion which is illustrated in Figure 1. Alazi and Ram (2018) suggested that due to variation in the composition of plant biomass, the mycozyme cocktail may also vary, therefore, it becomes even more important in industrial interest to produce desired mycozyme blends constitutively, independent of the substrate or the inducer used. Fungus must elaborate the significant amount of mycozymes to digest complex substrates and generate building block nutrients for absorption; the expense of secreting an enzyme must be balanced with the nutritional repay of the biosynthetic expenditure and growth rate. Fungi are capable of recognising the ligands available for nutrition. This is attained through the G-protein-coupled receptors (GPCRs), which are located at the plasma membrane comprising seven transmembrane domains and are majorly involved in environmental sensing and cell-signalling. GPCRs are turned on, when nutritional ligands bind to GPCR and trigger the GDP-GTP exchange on the Gα protein coupled with dissociation of Gα and Gβγ followed initiation of downstream signal cascades by Gα (Gao et al. 2021), adenylate cyclase shoots up levels of cyclic AMP (cAMP), which in turn trigger protein kinase A (PKA) downstream signalling cascades resulting in carbon catabolite repression. The apparatus of transcription regulation that exquisitely regulates the carbon catabolite repression system and secretion of lignocellulosic deconstruction enzymes of fungi can differ; some of these regulatory genes encoding extracellular mycozymes are described by Aro et al. (2005). Regulatory elements present in the promoter region have binding sites for the carbon catabolite repressor (CRE) gene, which encodes for plant cell wall degrading mycozymes. The inclusion of a deletion of the catabolite repressor gene, *cre-1*, in the triple β-glucosidase mutant of *Neurospora crassa* resulted in a strain that produced higher concentrations of extracellular active cellulases on cellobiose. Thus, the ability to induce cellulase gene expression using a common and soluble carbon source simplifies enzyme production and characterisation, which could be applied to other cellulolytic filamentous fungi (Znameroski et al. 2012). Following three measures have been suggested by Baker (2018) for overproduction of mycozymes: (1) evading nutritional repressor cues, (2) stopping transcriptional repression and (3) enhancing protein translation and secretion.

**Table 1.** Mycozymes used in various industries and their applications

Industry	Field	Mycozymes	Fungal species	Role in Industrial Processes	Reference
**Food processing**	Dairy	Peptidases	*Kluyveromyces lactis, Saccharomyces cerevisiae, Debaryomyces hansenii* and *Pichia anomala*	Accelerate cheese ripening	Klein et al. 2002
		Lipases	*Meyerozyma guilliermondii*	Low-cost production of acid using cheese whey as substrate	Knob et al. 2020
		β-Galactosidases	*Aspergillus lacticoffeatus*	Lactose removal in milk for synthesis of novel prebiotics	Beatriz et al. 2017
		Proteases	*Schizophyllum commune*	Environment friendly clean-in-place in dairy industry	Boyce and Walsh 2012
	Bakery	Amylases	*Rhizomucor miehei* CAU432	Increase bread volume and softness, colour and flavour and prevent staling	Wang et al. 2020b
		Xylanases	*Trichoderma stromaticum*	Improvement of dough properties, reduce water content in pasta	Almeida Carvalho et al. 2016
		Glucose oxidase	*Cladosporium neopsychrotolerans* SL16	Increase gluten strength, increase dough volume, texture and stability	Ge et al. 2020
		Cysteine Proteases	*A. oryzae*	Amelioration of cocoa organoleptics in biscuits & cookies	Murthy et al. 2020
		Prolyl-endoprotease	*A. niger*	Cleaves proline glutamine in gluten and thus used for making low immunogenic pasta for gluten sensitive population	Kumara et al. 2019
	Beverage (juice)	Pectinases	*Geotrichum candidum* AA15	Degradation of pectin in fruits to decrease viscosity and clarification of juice	Ahmed et al. 2020
		Glucoamylase	*A. flavus* NSH9	Starch breakdown for industrial processing application	Karima et al. 2019
		Laccase	*Trametes versicolor*	Increases the oxidative stability of edible vegetable oil	Guerberoff and Camusso 2019
	Meat and Fish	Aspartic Proteases	*Rhizomucor miehei*	Meat tenderisation, production of fish protein hydrolysates, viscosity reduction, skin removal and roe processing	Sun et al. 2018
		Lipases	*Candida rugosa*	Conversion of non-polyunsaturated fatty acid to polyunsaturated fatty acid by removing glycerol backbone of triglycerol in kilka fish oil	Hosseini et al. 2018
		Transglutaminase	*Pichia pastoris* GS115 expressing microbial transglutaminase	Restructure pork and crosslinking of soy protein isolate with chicken myofibrillar protein increased hardness and chewiness	Yang and Zhang 2019
	Beer and Wine	Amylases	*Monascus purpureus, Rhizopus oryzae, Pichia guilliermondii, Saccharomycopsis fibuligera* and *Saccharomyces cerevisiae*	Hydrolysing starch during traditional brewing of Wuyi Hong Qu glutinous rice (wine)	Xu-Cong et al. 2015
		Glucanases	*A. awamori*	Hydrolysing glucans to reduce viscosity and improve filterability (beer)	Liu et al. 2020a
		Cellulases, Hemicellulases	*Lichtheimia ramosa*	Accelerate cell wall digestion in grains and saccharification of sugarcane bagasse (beer)	Garcia et al. 2018
		Xylopectinases	*Mucor* sp.	Breakdown pectin of brewer’s spent grain to accelerate pre-fermentation stage and enhance clarification (beer)	Hassan et al. 2020
	Prebiotic functional food	Endoglucanase 1	*Trichoderma reesei* expressed in *P. pastoris*	simultaneous production of XOS and COS	Tao et al. 2019
		Xylanase	*T. asperellum* ND-1 in *P. pastoris*	hydrolytic activity towards corncob xylan produced 50.44% of xylobiose within 0.5 h	Zheng et al. 2020
		Mannnanase	*A. terreus*	Generates MOS from locust bean gum, guar gum and konjec gum	Jana and Kango 2020
		Inulinase	*Aspergillus tritici* BGPUP6	FOS yield (19.40%) containing 3.70% ketose (GF2), 2.71% nystose (GF3) and 1.42% fructofuranosyl nystose (GF4) were obtained from inulin (10%)	Singh et al. 2020
		Chitosanase	*Beauveria* *bassiana* expressed in *P. pastoris* GS115	Chitosan oligosaccharides (GlcN)5 and (GlcN)6 were completely degraded by BbCSN-1, thus its application in production of chitosan oligosaccharides	Liu et al.2020b
**Animal feed**		Phytase	*Acremonim zeae*	Degrading phytic acid to release phosphorus under alkaline conditions and used for pig diet	Pires et al. 2019
		Cellulases, Hemicellulases	*Chrysoporthe cubensis*	Potential in biomass saccharification processes	Falkoski et al. 2013
		β-Glucanases	*Phialophora* sp. G5	Removal of β-glucans, reducing viscosity of barley-bean feed and mash and increased filtration rate of mash	Zhao et al. 2012
**Starch processing (including food, pharma and ethanol)**	Food	Amylases	*Rhizomucor miehei*	Starch saccharification to produce high-maltose syrup and improved quality of bread	Wang et al. 2020b
	Pharma	Glucoamylase	*A. niger*	Developing starch base nanocrystals as natural carriers for nutraceutical delivery	Hao et al. 2010
	Ethanol	Amylase	*Rhizopus oligosporus*	Ethanol biosynthesis by fast hydrolysis of cassava bagasse	Escarambonia et al. 2018
**Technical application**	Pulp and paper	Cellulases	*A. niger* MK543209	Fibre modification (improving softness), de-inking in paper recycling for bioethanol production	Darwesh et al. 2020
		Xylanases	*Talaromyces thermophilus*	Enhancing pulp bleaching process efficiency and releases chromophores and reduced sugars	Maalej-Achouri et al. 2012
		Cellulase, xylanase, Laccase and Lipases	*Trichoderma viride* VKF-3, *Fusarium equiseti* MF-3 and *A. japonicus* MF-1 respectively	Pitch control in pulping process	Kumar et al. 2018
	Textile	Cellulases	*Trichoderma reesei* ATCC 24449	Textile waste valorisation	Wang et al. 2018
		Amylases	*Aspergillus* sp.	Removal of starch coating (desizing)	Aggarwal et al. 2019
		Pectinases	*A. tamarii*	Bioscouring and phytopigments processing	Shanmugavel et al. 2018
		Laccases	*Phomopsis sp.*	Bleaching and deinking and biotransformation of aniline blue	Navada and Kulal, 2020
		Cold-active Lipases	*Penicillium canesense* BPF4 and *Pseudogymnoascus roseus* BPF6	Lipid stain removal and facilitating cold washing as a step towards mitigation of climate change	Sahay and Chouhan 2018
		Serine alkaline Proteases	*Penicillium chrysogenium* X5	Protein stain removal	Omrane Benmrad et al. 2018
	Biodiesel and bioethanol	Lipases	*Beauveria bassiana* expressed in *Aspergillus nidulans* A773	Transesterification of triglycerides (biodiesel)	Spiropulos Gonçalves et al. 2020
		Cellulases	*A. oryzae* MDU-4	Cellulose hydrolysis in lignocellulosic ethanol production	Saini et al. 2020
		Xylanases	*Thermomyces lanuginosus* and *Trichoderma reesei*	Degradation of hemicellulose	Juodeikiene et al. 2011
		Laccases	*Ganoderma lucidum* MDU-7	Digestion of lignin waste	Saini et al. 2020
	Leather	Lignolytic enzymes	*Trametes villosa* SCS-10	Removal of unwanted fats and dyes during soaking and liming process and treatment of wastewater	Ortiz-Monsalve et al. 2017
**Therapeutic industry**	Oncolytic enzymes	Asparaginase	*A. terreus*	Efficient anticancerous drug against lung cancer cell lines A549	Baskar et al. 2018
	Biosensor kits	Pyranose oxidase	*Trametes ochracea* POx variant L545C	Useful in clinical biochemistry for measuring blood glucose better than glucose oxidase	Abrere et al. 2020
	Anti-inflammatory	Feruloyl esterase	*A. niger*	Ferulic acid shows an anti-inflammatory and antioxidant capability	Zhi Na et al. 2019
	Antioxidant	Cu/Zn Superoxide dismutase	*A. niger* 26	Antioxidant properties of superoxide dismutase helps in scavenging reactive oxygen species	Dolashki et al. 2008
	Dietary supplement	β-galactosidase	*A. niger* F0215	Makes dairy products suitable for consumption of lactose intolerant patients	Dandan et al. 2017
**Cosmetic and personal care**		Alkaline protease	*Neocosmospora* sp. N1	Blood stain removal and produces cleaner, whiter, smoother skin as compared to sulphide treatment	Matkawala et al. 2019
		Laccase and Tyrosinase	*T. versicolor* and *A. bisporus respectively*	Cross-linkage of collagen with laccases and tyrosinase act as tanning agent	Jus et al. 2011
		Superoxide dismutase and Glutathione reductase	*Chaetomium globosum*	Antioxidant activity is applicable in health industry	Gao et al. 2012
		Lignin peroxidase isozyme H8; glucose oxidase	*Phanerochaete chrysosporium*; *A. niger*	Melanin decolourisation with in-situ generated H2O2 for whitening application of cosmetics	Sung et al. 2011
		Palatase 20,000 L, Lipase AYS “Amano”, Lipase A “Amano” 12, Piccantase A and Piccantase AN	Five commercial fungal lipases	Lipase-catalysed synthesis of natural aroma-active 2-phenylethyl esters in coconut cream	Hui Shan et al. 2011

Fungal protein secretion has been delved at all stages, from protein targeting to endoplasmic reticulum (ER) to secretion and subsequent degradation by proteases with the goal of improving titre, rate and yield of target proteins. Generally, proteins secreted by fungi are glycosylated and many of them are associated with the cell envelope, the plasma membrane, and the cell wall. Deshpande et al. (2008) studied the N-glycosylation pathway in the cytoplasm and ER and found that it was highly conserved evolutionarily across all the filamentous fungi considered. Finally, enzymes are secreted out from the surface of the plasma membrane into the periplasmic space, where they may be subsumed into the cell wall or, in many instances, may be discharged across the cell wall into the external medium. Before proteins are secreted, they undertake sundry of post-translational modifications. These start in the ER and perpetuate as the proteins travel through the Golgi apparatus. Presumably, three changes may happen to a protein molecule: (1) proteolytic cleavage of zymogenic form to remove the signal sequence from a propeptide sequence, if present; (2) a folding process involving the formation of disulphide bridges to develop the tertiary and quaternary structures of the protein; and (3) glycosylation (Peberdy 1994). After proper folding and glycosylation, enzymes are secreted extracellularly. On the other hand, the unfolded protein response (UPR) and ER-associated protein degradation (ERAD) are in-charge for managing the peptides with incorrect folding (Bernasconi and Molinari 2011; Wang et al. 2014). The UPR checks the presence of unfolded proteins in ER and initiates the biosynthesis of chaperones and folding enzymes, whereas the ERAD lyses the misfolded proteins. Lastly, the passage of the proper folded protein vesicles to the Golgi apparatus by fusion with target membrane and its secretion to the extracellular environment occurs (Wang et al. 2020b).

## 2. Screening strategies for mycozymes

The booming demand for environmentally benign industrial processes relies on the ability of finding a biocatalyst suitable to ideal process conditions. Detection of enzyme activity depends on a biochemical estimation that accounts for either the product formation or substrate depletion (Sheludko and Fessner 2020). Identification of native fungal isolates producing glycosyl hydrolases is extremely important, because of the increased demand for these mycozymes in many industries. Some of the classical methods of screening employ the polysaccharide (cellulose, xylan, mannan etc.) in solid medium and after the development of the fungal colony, Congo red or suitable polymer binding dye, is used to create a visible halo of the zone of hydrolysis. Prajapati et al. (2018) screened cellulase production in *Aspergillus tubingensis* NKBP-55 by growing it on Czapek-Dox medium supplemented with (1%) carboxy-methyl cellulose as the sole C-source and observed clear zones of hydrolysis after staining with 1% (w/v) Congo red. Similarly, xylanase production in *Aspergillus* and *Trichoderma* and mannanase production in *Malbranchea cinnamomea, Melanocarpus albomyces, Aspergillus terreus, Myceliophthora thermophila* was detected using xylan and mannan supplemented medium followed by staining with Congo red and de-staining with 1% NaCl solution (Ramanjaneyulu et al. 2015; Ahirwar et al. 2017). Dye–polysaccharide inter-actions which provide a visual indication of polymer hydrolysis (clear zones or halos) have been used for decades. Screening of various mycozymes based on agar-plate assays has been listed in Table 2.

**Table 2.** Agar-plate assays for screening of various mycozymes

Mycozymes	Fungal species	Medium	Screening (plate assay)	Reference
**Protease** EC 3.4.21.112	Filamentous fungi (*Penicillium, Aspergillus, Cenococcum, Cochliobolus* and *Rhizopus*)	M medium (10% of lactose-free skimmed UHT milk); P medium (1% of dried plasma); R medium (1% of dehydrated red blood cells)	Formation of proteolysis halo	Schuster et al. 2019
**L-Asparaginase** EC 3.5.1.1	*Aspergillus terreus* MTCC 1782	Modified Czapek–Dox (MCD) agar plates with L-asparagine (10 g/L) as a sole nitrogen source. 0.009% phenol red dye and 0.007% of bromothymol blue BTB	Colour change from yellow to pink case of phenol red dye and yellow to blue for bromothymol blue dye	Kruthi and Devarai 2016
**Lipase** EC 3.1.1.3	*Penicillium* sp. *Aspergillus* sp.	Medium containing tributyrin (0.1%)	Clear hydrolytic halos	Griebeler et al. 2011
**Esterase** EC 3.1.1.2	*Fusarium oxysporum*	Triacetin agar (containing the colourant Rodamin B) culture media	Formation of a fluorescent halo around a colony	Gabriela and Tatiana 2005
**α-Amylase** EC 3.2.1.1	*Aspergillus niger*	Starch agar medium	Clear zones of hydrolysis on starch agar plates after pouring Iodine solution	Veerapagu et al. 2016
**Cellulase** EC 3.2.1.4	*Aspergillus tubingensis*	Mandel’s mineral medium with 1% Carboxymethyl cellulose	Clear zones ofhydrolysis after staining with 0.1% Congo – Red and de-staining with 1 N NaOH.	Prajapati et al. 2018
**Xylanase** EC 3.2.1.8	*Aspergillus* and *Trichoderma*	Mineral Salts Medium MSM-xylan medium (birchwood xylan,0.1%)	Clear zones of hydrolysis after plates flooded with 0.01% of congo red Solution and de-stained with 1% NaCl	Ramanjaneyulu et al. 2015
**Mannanase** EC 3.2.1.78	*Malbranchea cinnamomea* NFCCI 3724, *Melanocarpus albomyces, Aspergillus terreus, Myceliophthora thermophila* NFCCI 3725	0.5% mannan (LBG) agar medium	Clear zones of hydrolysis after staining with 0.1% solution of Congo red and de-stained with 1% NaCl solution	Ahirwar et al. 2017
**Pectinase** EC 3.2.1.15	*Aspergillus niger*	Pectinase screening agar medium (PSAM)	A clear halo zone around the colonies after plates were flooded with 50 mM Potassium iodide-iodine solution	Oumer and Abate 2018
**β-Galactosidase** EC 3.2.1.23	*Aureobasidium pullulans* NCIM 1050, *Aspergillus oryzae* NCIM 1212, *A. niger* NCIM 616, and *A. flavus* MTCC 9349	50 μL of X-gal (5-bromo-4 chloro-3 indole-β-D galactopyranoside) with 20 mg/mL in DMSO as inducer in the agar plates	Fungal colonies producing β-galactosidase appeared blue in colour	Panesar et al. 2016
**Inulinase** EC 3.2.1.7	*Aspergillus* sp., *Penicillium* sp., *Geomyces pannorum* DSM 62116, *Cladosporium cladosporoides* DSM 62121, *Sclerotinia sclerotiorum* DSM 1946	Medium containing inulin as the sole carbon source	Visual analysis of the mycelium formed after incubation for 144 hrs	Regina Gern Sandra et al. 2001
**Fructosyltransferase (FTase)** EC 2.4.1.9	*Aspergillus tamarii*	Czapek Dox agar	Glucose oxidase-peroxidase (GOD-POD) reagent coupled with 4-aminoantipyrine dye	Choukade and Kango 2019
**Chitinase** EC 3.2.1.14	*Paecilomyces lilacinus* (EF183511)	PDA plate with two pieces of shrimp shell	Degradation of shrimp shells was visualised after 7–10 days of incubation	Homthong et al. 2016
**Phytase** EC 3.1.3.8	*A. niger*	Phytase screening turbid agar media plates (PSM) containing 0.1% Na- phytate	Clear hydrolytic halos	Mittal et al. 2011
**Laccase** EC 1.10.3.2	*Penicillium chrysogenum*	PDA agar plates containing 3 mM of ABTS, 4 mM of guaiacol and 4 mM of tannic acid as substrates	ABTS screening-green zone formed, guaiacol screening-reddish brown oxidation zone and tannic acid screening dark brown oxidation zone	Senthivelana et al. 2019

Addition of colour changing indicator dyes can enhance the visibility during screening process. Kruthi and Devarai (2016) used modified Czapek–Dox (MCD) agar plates with L-asparagine (10 g/L) as a sole nitrogen source and used indicator dye, phenol red (0.009%) or bromothymol blue (0.007%), to observe colour change from yellow to pink or red and yellow to blue, respectively. Hydrolysis of synthetic substrates in the culture media, which is executed by the cleavage of the moiety attached to the substrate thus, producing a visible effect has also been used in detection of mycozymes. Panesar et al. (2016) screened β-galactosidase from fungi like *Aureobasidium pullulans* NCIM 1050, *Aspergillus oryzae* NCIM 1212, *A. niger* NCIM 616, and *A. flavus* MTCC 9349 using 50 μL of X-gal (5-bromo-4-chloro-3-indole-β-D galactopyranoside) in the solid medium with 20 mg/mL DMSO as inducer and observed the presence of β-galactosidase in fungal colonies appearing blue in colour. Use of modified synthetic substrates is a specific, precise and rapid method to detect desired mycozyme from among the consortia secreted by the fungus. In this, the substrate is modified by addition of synthetic signalogenic moiety and on enzymatic reaction, the signalophore conjugate is released from the synthetic substrate delivering a measurable signal. Allison et al. (2018) used fluorogenic soluble synthetic substrates like 7-amino-4-methylcoumarin (AMC) for detecting leucine aminopeptidase (LAP) and 4-methyumbelliferone (MUB) for ascertaining the presence of hydrolytic enzymes in the culture filtrate of *Neurospora discrete*. The fluorescence for hydrolytic enzymes was read at 365 nm/450 nm excitation/emission and for oxidative enzymes, absorbance was read at 410 nm. In recent years, there has been tremendous interest in the development of enzyme assays in connection with the high-throughput screening of enzymes for use in biocatalysis and drug discovery.

## 3. Production strategies of mycozymes

Fungi have the natural capability of colonising complex organics at low water activity level (a_w_). Occurrence of fungi in litter, wood logs, tree barks clearly suggests that these are adapted to grow on solid surfaces. In recent times, solid-state fermentation (SSF) has become an alternative industrial production system to produce enzymes and some of these important industrial mycozymes are listed in Table 3. SSF is a type of industrial fermentation with any microbial cultures’ growth on the surface of substrate or interior of the solid matrix in the absence of free-flowing water. Besides the koji-type systems, SSF cultures are now routinely used by researchers on other solid natural substrates, generally, agro-waste residues composed of lignocellulosic materials, like soybean hulls, wheat bran, rice straw, orange peels, red gram husk, soybean husk, rice stalk, sugarcane bagasse etc. (Dong-sheng et al. 2017; Lópeza et al. 2018; Mandari et al. 2019; Bruno et al. 2019; Melnichuk et al. 2020), but sometimes industrial waste of dairy, brewery, paper, pulp, and wood processing industries is also used as substrate for mycozyme production. For example, chicken feather meal for keratinase production by *Trichoderma harzianum* (Bagewadi et al. 2018) and maize distillery dried grain solubles (DDGS) for phytase production from *Trichoderma atroviride* (Pradoa et al. 2019). Thus, SSF is a technique to utilise organic wastes as raw materials to produce mycozymes, which could further be used for various industrial processes and at the same time contributing to solve the environmental issues caused by their inadequate disposal.

**Table 3.** Production of mycozymes in solid state fermentation (SSF)

Mycozyme	Fungus	Substrate	Culture Conditions			Activity	Reference
			pH	Temp.	Time		
**Alkaline Protease**	*Neurospora crassa*	soybean okara	5.0	30°C	72 hours	1959.82 U/g	Zheng et al. 2019
**Aminopeptidase**	*Aspergillus niger*	Orange peels, soybean hulls	7.05	30°C	5 days	1000 U/mL	Lópeza et al. 2018
**Keratinase**	*Trichoderma harzianum*	chicken feather meal g/20 ml, yeast extract 0.2%, glucose 0.9%	8.5	37ºC	10 days	10150 U/g	Bagewadi et al. 2018
**Protease**	*Aspergillus oryzae* and *Aspergillus flavipes*	wheat bran in water 1:1 (w/v)	-	30°C	120 hours	20.4 U/ mL	Pradoa et al. 2018
**L-Asparaginase**	*Aspergillus niger*	Passion fruit peel flour	-	25°C	24 hours	3746 U/gds	Da Cunhaa et al. 2018
**Lipase**	*Aspergillus niger*	*Prosopis juliflora*, red gram husk and cotton seed cake	7.0	35°C	72 hours	269.87 U/gds	Mandari et al. 2019
**feruloyl Esterase**	*Aspergillus niger*	De-starched wheat bran	-	-	72 hours	11984.1 U/gds	Gopalan et al. 2016
**α-Amylase**	*Aspergillus oryzae*	45% soybean husk and 55% flour mill by-product, without pretreatment	-	30°C	6 days	5560 ± 70 U/mL	Melnichuk et al. 2020
**Glucoamylase**	*Aspergillus awamori*	Waste bread pieces	-	-	40 hours	130.8 U/g	Melikoglu et al. 2015
**Endoglucanase**	*Trichoderma hamatum* *Aspergillus tubingensis*	Saw dust, paddy straw, cow dung, banana peel and wheat bran Copra Meal	6.0 5.0	37°C 30°C	6 days 5 days	55.3 ± 2.8 U/g 740.8 U/gds	Marraiki et al. 2020 Prajapati et al. 2018
**Exoglucanase**	*Aspergillus niger*	30.0 g wheat bran powder, 10.0 g rice stalk powder; sea water	5.0	50°C	30 min	7.10 U/mg protein	Dong-sheng et al. 2017
**β-glucosidase**	*Penicillium citrinum*	rice straw, rice bran, sugarcane bagasse	6.0	30°C	4 days	57.5 U/g	I-Son et al. 2010
**Xylanase**	*Fusarium solani*	Xylan, yeast extract	5.0	-	120 h	10.65 U/mg	Martínez-Pachecoa et al. 2019
**Mannanase**	*Trichoderma longibrachiatum* *Aspergillus oryzae*	rice straw Copra meal	7.0--	30°C 28°C	10 days 5 days	71.277U/g 434.6 U/gds	Hassan et al. 2019 Jana et al. 2018
**Pectinase**	*Aspergillus niger*	Fresh orange pomace ammonium sulfate yeast extract (1:1)	-	-	100 h	55 U/g	Mahmoodi et al. 2019
**α-Galactosidase**	*Aspergillus awamori*	wheat bran with defatted soy flour	5.0	28°C	120 h	75.11±0.29 U/g	Vidya et al. 2020
**β-Galactosidase**	*Aspergillus awamori*	wheat bran with defatted soy flour	5.0	28°C	120 h	155.34±1.26 U/g	Vidya et al. 2020
**β-Glucanase**	*Metarhizium anisopliae*	Sugarcane bagasse	-	28°C	14 days	11.54 ± 6.96 U/g	Bruno et al. 2019
**Inulinase**	*Mucor circinelloides*	Apple pomace and dahlia tubers powder in 9:1	6.4	30ºC	5.8 days	411.3 IU/gds	Singh et al. 2019
**Pullulanase**	*Aspergillus* sp.	Wheat bran	-	28ºC	-	396.2±1.33U/gds	Naik et al. 2019
**β-fructofuranosidase**	*Aspergillus tamarii*	Soy bran	-	30°C	72 h	229.43±4.88 U/mL	Batista et al. 2020
**Chitinase**	*Metarhizium anisopliae*	Sugarcane bagasse	-	28°C	14 days	12.07 ± 0.50 U/g	Bruno et al. 2019
**Phytase**	*T. atroviride*	Maize DDGS (distiller dried grains with solubles)	-	30°C	120 h	2000 U/mg protein	Pradoa et al. 2019
**Manganese Peroxidase**	*Phanerochaete chrysosporium*	cassava residue	-	30°C	6 days	186.38 nkat/g	Huixing et al. 2014
**Laccase**	*Lentinus tigrinus*	milled pistachio shell	-	30°C	-	172.0 U/mg	Sadeghian-Abadi et al. 2019
**Tannase**	*Aspergillus niger*	spent coffee ground	-	32°C	18 days	260.39 U/g	Mansor et al. 2019
**Ellagitannase**	*Aspergillus niger*	Ellagitannins	-	30°C	36 hours	606.67 U/L	De la Cruz et al. 2017

Another type of SSF uses an inert support with absorbed liquid medium. The support can be of natural origin like sugarcane bagasse, or synthetics like polyurethane, amberlite or vermiculite etc. De la Cruz et al. (2015) carried out SSF using high-density polyurethane foam (PUF) impregnated with culture medium and spore solution to reach 70% moisture content. Fermentation was carried out at temperature 30°C with aeration rate 0.5 vKgm (air volume/kg material) for 36 h to produce 606.67 U/L of ellagitannase from *A. niger*. In this type of SSF, the product recovery from inert support is less cumbersome because of convenient extraction procedures and products are obtained with fewer impurities (Singhania et al. 2008), however, the costs of the inert support are higher than in the previous case.

Wang et al. (2018) utilised textile wastes as substrate for cellulase production *via* submerged fungal fermentation and *Trichoderma reesei* ATCC 24449 was selected for highest cellulase activity (18.75 FPU/g). Abd El-Rahim et al. (2020) produced pectinase from fungi isolated from flax retting liquor using pectin mineral salt broth, at 30°C with shaking at 120 rpm for 120 h. Among various fungi, *Aspergillus pulverulentus* F23 produced 25.78 pectinase Unit/gram fungal biomass. Lately, issues with submerged fermentations (SmF), such as high-energy consumption, additional requirement of water and effluent generation are limiting its applications. Academic as well as industrial researchers are again taking notice of the advantages of SSF such as less water and energy consumption, low costs and high productivity. Ali et al. (2019) used 10 g of milled pistachio shell (particles size < 500 μM) and 2 mL liquid medium (yeast extract 0.2 g/L, glucose 2 g/L and CuSO_4_ 0.625 g/L) to produce 172.0 U/mg of laccase from *Lentinus tigrinus*. Thus, mycozyme production using SSF is crucial in chemical, pharmaceutical and environmental industries, and also makes an important contribution in waste management and sustainable development (Chen 2013).

## 4. Some industrially relevant mycozymes

### 4.1 Proteases

Proteases (EC 3.4) catalyse hydrolytic reactions that degrade protein molecules down to peptides and eventually to free amino acids. They constitute another large and complex group of enzymes, which differ from each other in terms of substrate specificity, nature of active site and catalytic mechanism followed, as well as pH and temperature optima and heat stability. The specificity of proteases, in particular, is governed by the type of amino acid residue(s) in their catalytic site (Ramos and Malcata 2017). The physical and chemical parameters of protease from fungi have been widely studied and described. Due to its varied nature, proteases are used in food and feed, waste management, detergent and medical sectors.

Prominent fungal producers belong to the genera *Aspergillus, Penicillium, Rhizopus, Mucor*and*Thermomyces*. Peptidases of *Kluyveromyces lactis, Saccharomyces cerevisiae, Debaryomyces hansenii* and *Pichia anomala* accelerated cheese ripening (Klein et al. 2002). An aminopeptidase of *Thermomyces lanuginosus* expressed in *A. niger* strain HL-1 showed significant increase in the degree of hydrolysis of soy protein and removed more hydrophobic amino acids from the N-terminal region of the polypeptide to decrease the bitterness (Lin et al. 2019). Darío et al. (2018) reported production of 1000 U/mL aminopeptidase from *A. niger* using orange peels and soybean hulls as substrate for SSF. Boyce and Walsh (2012) used protease of *Schizophyllum commune* for environment friendly clean-in-place of protein waste in dairy industry. Recently, Murthy et al. (2020) reported cysteine proteases of *A. oryzae* for amelioration of cocoa organoleptics in biscuits, cookies and crackers. Kumara et al. (2019) produced prolyl-endoprotease from *A. niger* and used it for cleaving proline and glutamine moieties present in gluten and making low immunogenic pasta for gluten sensitive population. Meat tenderisation, production of fish protein hydrolysate, viscosity reduction, skin removal and roe processing were carried out using aspartic proteases of *Rhizomucor miehei* (Sun et al. 2017). An acidic protease of *Metschnikowia reukaufii* SAP6 was expressed in *E. coli* and the purified enzyme had milk-clotting activity (Jing et al. 2010). Yang and Zhang (2019) expressed transglutaminase in *Pichia pastoris* GS115 and used it to restructure pork and crosslinking of soy protein isolate with chicken myofibrillar protein which increased hardness and chewiness. *Aspergillus flavipes* produced 20.4 U/mL protease on wheat bran in SSF (Pradoa et al. 2019). A salt-tolerant glutaminase of *A. oryzae* expressed in *E. coli* was found suitable for brewing high quality soy sauce with a high L-glutamic acid concentration (Masuoa et al. 2004). Serine alkaline protease of *Penicillium chrysogenum* X5 was used for protein stain removal in textile industry (Omrane Benmrad et al. 2018). Alkaline protease of *Neocosmospora* sp. N1 was used for blood stain removal as it yields cleaner, whiter, smoother skin as compared to sulphide treatment (Matkawala et al. 2019). De Medeiros et al. (2016) reported use of keratinase of *Trichosporon loubieri* RC-S6 in dehairing for leather production. The heterologous expression of SUB3 keratinase of *Microsporum canis* expressed in *P. pastoris* KM71 H provided a valuable tool for addressing investigations on the role of this keratinase in the specific cellular immune response and on its use in vaccination trials in cats (Descamps et al. 2003). L-asparaginase of *A. terreus* acted as an efficient anti-cancer drug against lung cancer cell lines A549 (Baskar et al. 2018). Saeed et al. (2018) expressed L-asparaginase of *A. terreus* in *E. coli* for treatment of acute lymphoblastic leukaemia. Katrolia et al. (2019) expressed protease of *Cordyceps militaris* CmFE in *P. pastoris* GS115 and used recombinant fibrinolytic enzyme as therapeutic agent for thrombolysis.

### 4.2 Cellulases

Cellulose is the most abundant renewable carbohydrate on the earth and the major constituent of plant cell wall. Cellulose is naturally embedded with lignin – hemicellulose matrix within the plant cell wall. It is being explored widely for the generation of fermentable sugar for bio-ethanol generation. Biofuel generation from cellulosic biomass utilises three steps *viz*. pre-treatment, enzymatic saccharification and ethanolic fermentation. After pre-treatment, generation of monosugars from the complex lignocelluloses is catalysed by cellulases and hemicellulases. Fermentation of released sugars is carried out by yeast for bio-ethanol production. Cellulose is a homopolymer composed of glucose units linked by β- 1,4 -glycosidic bonds. α-1,6-glycosidic bonding between glucose moieties and hydrogen bonding among cellulose fibrils gives rise to a compact crystalline structure which is difficult to digest by a single hydrolase. Cellulases represent a complex group of synergistically acting enzymes. They principally contain endo-1,4-glucanase (EC 3.2.1.4), which cleave randomly at internal amorphous cellulose sites causing rapid reduction in the cellulose DP while liberating cello-oligomers in the process; cellobiohydrolases (EC 3.2.1.91) act progressively on crystalline cellulose and primarily attack the reducing ends of polymer to produce cellobiose or short chain oligosaccharides and later, β-glucosidase (E.C. 3.2.1.21) hydrolyses cellobiose to glucose monomers (Wahlström and Suurnäkki 2015). Cellulases have remarkable applications in various industries including pulp and paper, textile, laundry, biofuels and food and feed and brewing industry (Prajapati et al. 2018).

Among fungi, major industrially used production hosts for cellulase production include *Trichoderma, Penicillium* and *Aspergillus*. Recently, Darwesh et al. (2020) reported cellulase of *A. niger* MK 543209 for fibre modification (improving softness), deinking and bioethanol production. Wang et al. (2018) used cellulase of *T. reesei* ATCC 24449 for valorisation of textile waste in textile and laundry industries. Cellulases of *Trichoderma viride* were used for pitch control during pulping process in paper and pulp industries (Kumar et al. 2018). Recently, Tao et al. (2019) reported simultaneous production of xylooligosaccharides (XOS) and cello-oligosaccharides (COS) by recombinant endoglucanase I of *T. reesei* expressed in *P. pastoris*, showing a novel application of genetically modified mycozyme for maximum utilisation of natural biomass and fortification of food and feed with functional food ingredients.

### 4.3 Hemicellulases

Hemicelluloses, comprising the second most abundant part of the plant biomass, are diverse group of structural and storage polysaccharides. Xylan, mannan and other hemicelluloses make up to 30% of the dry weight of the wood. Their enzymatic hydrolysis using fungal hemicellulases has led to several industrial applications, *viz*., biobleaching; wastepaper deinking; fruit juice maceration; upgradation of feed, fodder, and fibres; and saccharification of biomass.

#### 4.3.1 Xylanases

Xylan is a structural polysaccharide of plant cell wall, with a high potential for conversion into useful end products using xylanases. It is a heteroglycan composed of a linear chain of xylopyranose residues bound by β (1 → 4) linkages, with a variety of substituents linked to the main chain by glycosidic or ester linkages. Endo-1,4-β-xylanases (EC 3.2.1.8) are most important for xylan hydrolysis as they initiate the degradation of xylan into XOS and xylose (Ramanjaneyulu et al. 2015). Due to the structural heterogeneity of xylan, complete hydrolysis of xylan requires combined action of endo-1, 4-β-xylanase, β-1,4-D-xylan-xylanohydrolase, β-xylosidase and some accessory enzymes (Kango et al. 2005).

Many microbes such as bacteria, fungi and actinobacteria are known to produce xylanolytic enzymes, however, filamentous fungi are the preferred and most explored xylanase producers among all (Basu et al. 2018; Kumar et al. 2018; Aaa et al. 2018). Among these, *Thermomyces, Trichoderma*and*Aspergillus* are the most exploited genera for xylanase production. *Thermomyces lanuginosus* (previously known as *Humicola lanuginosa*) has gained considerable interest due to its ability to produce high titres of thermostable endo-xylanase (Khangwal et al. 2020). Apart from being used in conjunction with cellulases for biofuel production, xylanases have numerous applications in various industries such as food and animal feed, paper and pulp processing, textiles etc. (Basu et al. 2018). Xylanase sourced from *Trichoderma stromaticum* improved the dough quality by reducing water content during pasta preparation (Almeida Carvalho et al. 2016). Similarly, Kumar et al. (2018) reported xylanase of *Fusarium equiseti* MF-3 showing potential in pulp and paper industry for pitch control in pulping process. Xylanases of *T. lanuginosus* and *T. reesei* have been used for enzymatic hydrolysis of xylan rich agro-waste for production of 2 G-bioethanol (Juodeikiene et al. 2011). Xylanases of *Talaromyces thermophilus* showed enhanced pulp bleaching process efficiency and released chromophores and reduced sugars (Maalej-Achouri et al. 2012). Recently, Bhardwaj et al. (2020) expressed xylanase gene, XynF1 of *A. oryzae* in *E. coli*. Cellulase-free xylanases are desirable for biobleaching where they replace chlorine based bleaching agents and thus, release of toxic organo-chloro compounds is avoided. Recently, Martínez-Pachecoa et al. (2019) optimised the xylanase production with low cellulase titres in *Fusarium solani* by SSF. Nowadays, nutritional quality of food and feed is being enhanced by augmenting them with functional food ingredients like XOS. Hydrolysis of xylan rich agro-waste products, (e.g. corn cobs) using fungal xylanase generates XOS and also leads to value addition and proper disposal of agricultural waste. Recently, Zheng et al. (2020a) provided a cost-effective method of producing XOS from corn cobs by expressing xylanase gene *Taxy11* of *Trichoderma asperellum* ND-1 in *P. pastoris*.

#### 4.3.2 Mannanases

Mannans, chiefly composed of mannose, are the plant storage and structural polysaccharides commonly known as gums. These being heteropolymeric require synergistic action of different enzymes for complete degradation including, β-mannanase (EC 3.2.1.78), β-mannosidase (EC 3.2.1.25), β-glucosidase (EC 3.2.1.21), α-galactosidase (EC 3.2.1.22) and acetyl esterase (EC 3.1.1.6). Several mannanolytic mycozymes are reported from the genera *Aspergillus* (Soni et al. 2016), *Penicillium* (Blibech et al. 2011) and *Trichoderma* (Elena et al. 2009). *Aspergillus aculeatus* endo-β-mannanase (Man1) and *Talaromyces emersonii* α-galactosidase (Agal) genes were co-expressed in *S. cerevisiae* Y294 and the maximum enzyme activity for Man1 (374 nkat/mL, pH 5.47) and Agal (135 nkat/mL, pH 2.37) were reported (Malherbe et al. 2014). β-Mannanases have extensive applications in industries such as food and feed processing. The N-glycosylation in the loop area intern improved thermal stability, pH stability and protease-resistance of the *Armillariella tabescens* β-mannanase (Hu et al. 2017). *Rhizopus microsporus* endo-β-mannanase was structurally characterised and it showed altered binding properties with different oligosaccharides (You et al. 2018). Mannanases from *Malbranchea cinnamomea, A. oryzae* and *A. terreus* that generate mannooligosaccharides (MOS) from locust bean gum, guar gum and konjac gum have been reported (Ahirwar et al. 2017; Li et al. 2017; Jana and Kango 2020).

#### 4.3.3 Pectinases

Pectin is another structural polysaccharide that occurs in the primary cell wall and intracellular layer of fruits, such as apples, oranges, lemons etc. and is characterised by the presence of galacturonic acid residues (Mudgil 2017). Pectinases (EC 3.2.1.15) constitute a heterogeneous group of enzymes that break down complex polysaccharides of plant tissues into simpler molecules like galacturonic acids either by depolymerisation (hydrolases and lyases) or de-esterification (esterases) (Singh et al. 2019). Pectinases are produced predominantly by *Aspergillus* and *Penicillium* and are used to accelerate rates of clarification and filtration to remove pectin from fruit base prior to gel formation during jam manufacture. *A. niger g*rown on fresh orange pomace in SSF produced exo-pectinases (55 U/g) and endo-pectinases (10 U/g) (Mahmoodi et al. 2019). Pectinase of *A. tamarii* was used for bioscouring and phytopigment processing in textile industry (Shanmugavel et al. 2018). Recently, Ahmed et al. (2018) produced pectinase from *Geotrichum candidum* AA15 and used it for the degradation of pectin in fruits to decrease viscosity and clarify juice. Pectinase of *Mucor* sp. was used to breakdown pectin of brewer’s spent grain to accelerate pre-fermentation stage and enhance clarification during beer production (Hassan et al. 2019). The recombinant NfPG4 and NfPG5 genes of *Neosartorya fischeri* expressed in *P. pastoris* GS115 were shown to be exo- and endo-polygalacturonases, respectively. Both pectinases were tolerant towards a wide range of pH, temperature, metal ions, making them a suitable choice for industrial applications (Li et al. 2015).

#### 4.3.4. Inulinase and Fructosyltransferase (FTase)

Inulins are made up of fructose units linked typically with a terminal glucose by glycosidic linkage. Inulin belongs to the fructan group of storage and transport polysaccharides and in many plant species it is synthesised from sucrose by adding a fructosyl unit (Choukade and Kango 2020). Commercially exploited prebiotic fructooligosaccarides, kestose (GF2), nystose (GF3), and β-fructofuranosylnystose (GF4) are produced from sucrose by fructosyltransferase (FTase) from plants, bacteria and fungi.

Inulinases (E.C. 3.2.1.7) are the key enzymes that take part in inulin metabolism in plants and microorganisms. They hydrolyse natural plant fructan inulin into fructose and inulooligosaccharides (IOS) upon acting on glycosidic linkages with terminal glucose (Kango and Jain 2011). Rawat et al. (2015) reported inulinase and FTase activity in some *Aspergilli* and *Penicillia*. Fructosyltransferase (EC 2.4.1.9) is known to hydrolyse sucrose and transfer fructosyl group to an acceptor molecule to generate fructooligosaccharides (FOS) along with glucose and fructose (Ganaie et al. 2014). FTase possesses transfructosylating activity, cleaves the β-1,2 linkage of sucrose and transfers fructosyl group to an acceptor molecule leading to formation of FOS and release of glucose.

Industrial production of FOS involves the action of enzymes with transfructosylating activity isolated from microbial sources like fungi such as *Aspergillus japonicus, A. niger, A. sydowii, A. foetidus, A. oryzae, A. pullulans, Penicillium citrinum, P. frequentans*, and *Fusarium oxysporum* (Bali et al. 2015). Jiang et al. (2016) isolated a novel yeast, *Aureobasidium* sp. P6, from a mangrove ecosystem producing 30.98 U/mL inulinase. The inulinase also had transfructosylating activity at higher concentration of sucrose (30%) leading to FOS production. Wang et al. (2016a) used an industrial strain, *A. niger* ATCC 20611, to enhance the production of FOS, wherein they have used polyethylene glycol (PEG)-mediated protoplast transformation system for strain improvement. The transformed *A. niger* ATCC 20611, exhibited a 58% increase in specific β-fructofuranosidase activity (up to 507 U/g), compared to the parental strain (320 U/g). Previously, Tanriseven and Aslan (2005) have also immobilised commercially available *A. aculeatus* FTase (Pectinex Ultra SP-L) in Eupergit C with efficacy of 96% and maintained the recycling up to 20 days to obtain GF4, GF3 and GF2 in FOS mixture. Immobilised enzyme also showed a higher temperature optimum at 65°C. Wang et al. (2016d) cloned an endo-inulinase in *S. cerevisiae* and deleted its sucrase gene which resulted into high content FOS production (~90%) from inulin in a single step. Recently, Bao et al. (2019) expressed *Lipomyces starkeyi* NRRL Y-11557 inulinase gene (INU3B) in *E. coli* for the production of nutraceutical FOS.

Production of an extracellular, thermostable inulinase was carried out by a newly isolated strain of *A. tubingensis* CR16 using wheat bran and corn steep liquor (CSL) in SSF. After parametric optimisation, the fungus produced 1358.6 U/g inulinase, showing 5-fold enhancement (Trivedi et al. 2012). Similarly, Singh et al. (2018) demonstrated the applicability of apple pomace as a potent substrate in SSF for inulinase production by *Mucor circinelloides.*

### 4.4 Amylases

Starch is the most abundant storage polysaccharide on the earth and major component of many staple crops such as, potato, wheat, corn and rice. Apart from being staple food such as bread or rice, it also finds use as a thickener and a gelling agent in food industry. Starch consists of linear insoluble amylose and branched soluble amylopectin. In amylose, glucose is linked by 1,4-glycosidic bonds in a linear fashion, while in amylopectin some of the chains are linked by α-1,6-linkages giving it a branched structure.

Amylases, including α-amylase and glucoamylase, are perhaps the most important enzymes in present day biotechnology due to their wide range of applications in numerous industrial processes, including food, fermentation, textiles, and paper industries (Parashar and Satyanarayana 2017). As mentioned earlier, α-amylase bears historical relevance from the point of view of industrial application of mycozymes. After the production of Taka-diastase in 1894 from *A. oryzae*, α-amylase was also used as a textile desizing agent in Japan in 1905. Later in 1959, *Rhizopus* sp. was used to produce glucoamylase. Amylolytic enzymes account for about 30% of total industrial enzymes (Vaidya et al. 2015).

## 4.4.1 α-Amylases

α-Amylases (EC 3.2.1.1) are extracellular endo-acting enzymes that randomly hydrolyse α-1,4 glycosidic bonds in starch to produce maltose and dextrins. Most industrial applications use *α-*amylases for saccharification or liquefaction purposes. Fungal sources of industrial *α-*amylases are mostly confined to *Aspergillus, Penicillium and Rhizopus* sp. (Li et al. 2011). *Aspergillus* being one of the prominent and notably the most explored genera for *α-*amylases, particularly, *A. oryzae* (Taka-diastase) and *A. niger α-*amylases have been used extensively in the starch industry. Aggarwal et al. (2019) reported amylase of *Aspergillus* sp. for removal of starch coating (desizing) from textile. Recently, Wang et al. (2020a) reported amylases from *R. miehei* CAU432 which can be used for increasing bread volume and softness, enhance colour and flavour and even in preventing staling of bread. Xu-Cong et al. (2015) produced α-amylase from *Monascus purpureus, R. oryzae, Pichia guilliermondii, Saccharomycopsis fibuligera* and *S. cerevisiae,* which were used for hydrolysing starch during traditional brewing of Wuyi Hong Qu glutinous rice for wine production. Similarly, ethanol biosynthesis by fast hydrolysis of cassava bagasse by amylase sourced from *Rhizopus oligosporus* has been demonstrated by (Escarambonia et al. 2018). However, being mesophilic, the enzymes are not thermostable and thus bacterial *α-*amylases replace them in the very first step of gelatinisation (or cooking) at high temperature. Wang et al. (2019a) expressed a thermostable α-amylase active at 80°C from *Thermomyces dupontii* (*Td*AmyA) in *P. pastoris* and have demonstrated its applicability in maltose syrup production.

### 4.4.2 Glucoamylases

Glucoamylase (EC 3.2.1.3) is an exo-acting enzyme that cleaves α-1,4 linkages from the non-reducing ends but can also cleave α-1,6 linkages at the branching point of amylopectin, thus leading to successive and complete degradation of starch into glucose. Most commercial glucoamylases are sourced from *Aspergillus* spp. (Carrasco et al. 2017). Recently, Fabiane et al. (2020) produced ethanol from rice by-products using amylases secreted by *Rhizopus microsporus* var. *oligosporus*. Melikoglu et al. (2015) used waste bread pieces for solid-state production of glucoamylase from *Aspergillus awamori*. Glucoamylase of *A. niger* was used for developing starch-based nanocrystals as natural carriers for nutraceutical delivery (Hao et al. 2010). *GA2* gene of *Aspergillus flavus* glucoamylase was expressed in *P. pastoris* GS115 and the mycozyme generated larger, deeper, holes on the starch granules of raw sago starch, indicating rGA2 is an excellent candidate for industrial starch processing applications (Karima et al. 2019).

### 4.5 Laccases

After cellulose and hemicelluloses, lignin is the most abundant component of plant residues, and is relatively recalcitrant. Also, the degradation rates of lignocellulosic materials are negatively correlated to their lignin content or to their lignin-to-N ratio. Lignin is an aromatic biomolecule that is degraded at a much slower rate than cellulosic and non-cellulosic polysaccharides and proteins. Laccases (EC 1.10.3.2) are multicopper biocatalysts that catalyse the oxidation of mainly phenolic compounds by one electron transfer with the concurrent reduction of oxygen to water. Laccase is prominently used in wastewater management, textile, pulp and paper industries. Among fungi, laccases are particularly abundant in the white-rot fungi, which are the only organisms which have ability to decompose the whole wood components (i.e. cellulose, hemicellulose and lignin) so far (Daljit and Rakesh 2009). The most studied fungus for laccase production is the white-rot fungus, *Trametes versicolor* (Rodrıguez-Couto 2018). Recently, laccase of *T. versicolor* was used for increasing the oxidative stability of edible vegetable oil (Guerberoff and Camusso 2019). Ortiz-Monsalve et al. (2017) utilised lignolytic mycozymes of *Trametes villosa* SCS-10 for the removal of unwanted fats and dyes during soaking and liming process and treatment of wastewater. Recently, Navada and Kulal (2019) reported laccase of *Phomopsis* sp. for bleaching, deinking and biotransformation of aniline blue in textile industry. Digestion of lignin waste using laccase of *Ganoderma lucidum* MDU-7 for production of bioethanol is relevant for management of waste from pulp and paper industry (Saini et al. 2020). Improved activity, stability at alkaline pH and its role in the improved dye decolourisation suggested the application potential of the recombinant laccase of *Coprinopsis cinerea* expressed in *P. pastoris* GS115 for wastewater treatment (Xu et al. 2018).

### 4.6 Chitinases

Chitin, found as ordered crystalline microfibrils in the structural component of crustaceans and insects, is the most abundant biopolymer in nature after cellulose (Verma and Fortunati 2019). Chitinases (EC 3.2.1.14) hydrolyse chitin, a β-(1→4) linked N-acetyl glucosamine structural polysaccharide (Pérez and Tvaroška 2014). Chitinases are produced by plants as part of their defence mechanism against the invading fungal pathogens; whereas, microbial chitinases, produced by bacteria (*Streptomyces* and *Bacillus* spp.) are secreted to assist in the breakdown and assimilation of fungal cell walls, whereas fungal chitinases assist fungal cell-wall morphogenesis; only in species of mycoparasitic fungi, such as *Trichoderma harzianum, Aphanocladium album* and *Gliocladium virens* to attack and degrade hyphae of other molds (Ramos and Malcata 2017). *Metarhizium anisopliae* produced 12.07 U/g chitinase on sugarcane bagasse in SSF (Bruno et al. 2019). Chitinases have wide-ranging applications including the preparation of pharmaceutically important chito-oligosaccharides and N-acetyl,D-glucosamine, preparation of single-cell protein, isolation of protoplasts from fungi, control of pathogenic fungi, treatment of chitinous waste, mosquito control and morphogenesis etc. (Hamid et al. 2013). Recently, Liu et al. (2020b) expressed chitosanase of *Beauveria bassiana* in *P. pastoris* GS115 and demonstrated its use in the production of chitosan oligosaccharides. Chitinase gene of *T. harzianum*, Chit46 expressed in *P. pastoris* GS115, proved to be a good candidate for the green recycling of chitinous waste and inhibiting the growth of the phytopathogenic fungus *Botrytis cinerea* (Jun-Jin et al. 2019). Seven chitinases from different bacteria and fungi were produced, characterised and their biocontrol abilities against graminaceous stem borers *Eldana saccharina, Chilo partellus* and *Sesamia calamistis* were assessed; out of which chitinase from the thermophilic *T. lanuginosus* SSBP (Chit1) was found to be more acid-stable than the bacterial counterparts and caused 70% mortality in star larvae of *E. saccharina* (Okongo et al. 2019). A potent chitin-hydrolysing enzyme from *Myrothecium verrucaria* (rMvEChi) has been shown to affect the growth and development of *Helicoverpa armigera* and control plant fungal pathogens (*Ustilago maydis* and *Bipolaris sorokiniana*) (Vidhate et al. 2019).

### 4.7 Lipases

Lipases (EC 3.1.1.3) catalyse the hydrolysis of long-chain triglycerides into glycerol and fatty acids. Lipases sourced from bacteria and fungi are relatively stable and are capable of catalysing a variety of reactions and thus, are potentially important for diverse industrial applications (Hou and Shimada 2009). *A. niger* when grown on *Prosopis juliflora*pods, red gram husk and cotton seed cake mixture in the ratio of (6.66:1.66:1.66) produced 269 U/gds lipase (Mandari et al. 2019). Multifarious industrial applications of fungal lipases in the detergent, bioremediation, food, flavour industries, biocatalytic resolution of pharmaceuticals, esters and amino acid synthesis, making of fine agrochemicals, biosensor, cosmetics and perfumery make them a very important enzyme (Hasan et al. 2006). Lipase from *Candida rugos*a was used for the conversion of non-polyunsaturated fatty acids to polyunsaturated fatty acids by removing glycerol backbone of triglycerol in kilka fish oil (Hosseini et al. 2018). Sahay and Chouhan (2018) demonstrated lipid stain removal by facilitating cold-washing as a step towards mitigation of climate change using cold-active lipases of *Penicillium canescens* BPF4 and *Pseudogymnoascus roseus* BPF6. Lipase of *Rhizopus chinensis* expressed in *P. pastoris* GS115 showed esterification of short-chain fatty acids with ethanol (Yu et al. 2009). Recently, Spiropulos Gonçalves et al. (2020) reported lipases of *B. bassiana* expressed in *Aspergillus nidulans* A773 and applied it for production of biodiesel by transesterification of triglycerides.

## 5. Cloning and expression of mycozymes

Although mycozymes have diverse potential applications and prominent presence, there are some limitations associated with them. First, it is difficult to obtain in-depth understanding of the biocatalytic action of the mycozyme in a mixture. Second, an accountable production economy can be challenging to obtain, as it may be difficult to optimise the production of a specific mycozyme without knowing the target gene. Third, the mixture may consist of proteases which can hamper the activity of the specific mycozyme in need. The recombinant mycozymes can be produced in high yields thus providing new tools for functional studies through careful selection of the expression system in the substantially higher purity. Wang et al. (2019a) expressed α-amylase gene of *Thermomyces dupontii, Td*AmyA in *P. pastoris* GS115 with AOX1 promoter and it produced the highest maltose content of 51.8% after 8 h hydrolysis indicating application in maltose syrup production. The expression system allowed high level α-galactosidase production in medium with glucose as the sole carbon source and without a requirement for an inducer with a yield of 2.45 U/mL, which is nearly 3-fold higher than the yield obtained from *A. fumigatus* grown in locust bean gum containing medium (Gürköka et al. 2009). During the last few years expression cloning has been applied to several fungal enzymes and has proven very efficient in cloning of mycozyme genes. Recently, Bhardwaj et al. (2020) expressed xylanase of *A. oryzae* in *E. coli* BL21 which exhibited a wide range of activity at different pH (3.0–10.0) range and temperature (30–70°C) with an optimum pH and temperature as 5.0 and at 30°C, respectively, making it useful for a variety of industrial applications. The superior properties of mannanase of *Aspergillus kawachii* expressed in *P. pastoris* X33, strongly facilitates MOS preparation and application in food and feed area (Liu et al. 2019). Some of the industrially relevant mycozymes which are cloned and expressed in a heterologous hosts are listed in Table 4. Fatimi et al. (2018) expressed cellobiohydrolase of *Trichoderma virens* in *A. niger* strain PY11 with *Gla*Pr as the promoter gene which enhanced the CbhI activity towards Avicel (0.011 U/mg), which could be useful in complex biomass degradation. *Penicillium griseoroseum* PG63 efficiently expressed phytase of *P. chrysogenum* with an increase of up to 5.1 times in the enzymatic activity and stability profiles. The ability to release inorganic phosphate from cereals which are commonly used for pig feed suggested the potential application of phytase produced by *P. griseoroseum* T73 in the animal nutrition industry (Ribeiro Corrêa et al. 2014). Furthermore, the new enzyme backbones may contribute significantly to a better understanding and determination of amino acid residues that may be of importance for the enzymatic characteristics.

**Table 4.** Heterologous expression of Mycozymes in various hosts

Mycozymes	Fungal source	Gene	Plasmid Vector	Promoter	Expression Host	Applications	(Reference)
**Protease**	*Cordyceps militaris*	CmFE	pPIC9K	*AOX*1	*P. pastoris* GS115	Recombinant fibrinolytic enzymes as therapeutic agents for thrombolysis.	(Katrolia et al. 2019)
**Aminopeptidase**	*Thermomyces lanuginosus*	The	pMD20	PnaII/TPI	*A. niger* strain HL-1	Significantly increase the degree of hydrolysis of soy protein and remove more hydrophobic amino acids from the N-terminal region of the polypeptide to decrease the bitterness.	(Lin et al. 2019)
**Renin**	*Metschnikowia reukaufii*	SAP6	pET-24a	T7	*E. coli* BL21 (DE3)	Milk-clotting	(Jing et al. 2008)
**Lipase**	*Rhizopus chinensis*	rcl	pPIC9K	AOX1	*P. pastoris* GS115	Very high ability of esterification of short-chain fatty acids with ethanol.	(Yu et al. 2008)
**Esterase**	*Thermomyces lanuginosus*	tle	pET28	T7	*E. coli* BL21 (DE3)	For flavor development in food and alcoholic beverages	(Li et al. 2014)
**α-Amylase**	*Thermomyces dupontii*	*Td*AmyA	pPIC9K	AOX1	*P. pastoris* GS115	Maltose syrup production.	(Wang et al. 2019a)
**Glucoamylase**	*Aspergillus flavus*	GA2	pPICZαC	AOX1	*P. pastoris* GS115	Prolonged incubation generated larger, deeper, holes on the starch granules of raw sago starch, indicating its industrial starch processing applications.	(Karima et al. 2019)
**Endoglucanase**	*Aspergillus fumigatus*	Cel7	pKLAC2	PLAC4-PBI	*K. lactis* GG799	Application in the paper industry and in saccharification processes for the production of biofuel and other chemicals using cellulosic materials.	(Rungrattanakasina et al. 2018)
**Cellobiohydrolase**	*Trichoderma virens*	cbhI	ANIp5MS	*Gla*Pr	*A. niger* strain PY11	Useful in complex biomass degradation.	(Fatimi et al. 2018)
**Xylanase**	*Aspergillus oryzae*	XynF1	pET28a	T7	*E. coli* BL21 (DE3)	Pulp biobleaching in paper industry	(Bhardwaj et al. 2020)
**Mannanase**	*Aspergillus kawachii*	ManAK	pPICZαA	AOX1	*P. pastoris* X33	Mannooligosaccharide (MOS) preparation and application in food and feed area.	(Liu et al. 2019)
**Pectinase**	*Neosartorya fischeri*	NfPG4 and NfPG5	pPIC9	AOX1	*P. pastoris* GS115	The recombinant NfPG4 and NfPG5 were shown to be exo- and endo-polygalacturonases, respectively. Both enzymes were tolerant against a wide range of pH, thermostable, and resistant to many metal ions or chemicals, making them an interesting candidate for industrial applications with a preference for thermophilic pectinases.	(Li et al. 2015)
**α-Galactosidase**	*Aspergillus fumigatus*	aglB	pAN52-4	gpdA	*Aspergillus sojae*	The expression system allowed high level α-galactosidase production in media with glucose as the sole carbon source and without a requirement of an inducer with a yield of 2.45 U/mL which is nearly 3-fold higher than the yield obtained from *A. fumigatus* grown in locust bean gum containing medium.	(Gürköka et al. 2009)
**β-Galactosidase**	*Paecilomyces aerugineus*	PaGalA	pPIC9K	AOX1	*P. pastoris* GS115	The extremely high expression levels coupled with favourable biochemical properties make this enzyme highly suitable for commercial purposes in the hydrolysis of lactose in milk or whey.	(Katrolia et al. 2011)
**β-Glucanase**	*Coprinopsis cinerea*	eng16A	pPICZαA	AOX1	*P. pastoris* GS115	Hemicellulose degradation	(Wang et al. 2016a)
**Inulinase**	*Lipomyces starkeyi*	INU3B	pET22b	T7	*E. coli* BL21 (DE3)	Industrial production of FOS	(Bao et al. 2019)
**Chitinase**	*Trichoderma harzianum*	Chit46	pPIC9K	AOX1	*P. pastoris* GS115	It’s a good candidate for the green recycling of chitin waste. It could also significantly inhibit growth of the phytopathogenic fungus *Botrytis cinerea*, which endowed it with the potential as a biocontrol agent.	(Jun-Jin et al. 2019)
**Phytase**	*Penicillium chrysogenum*	phy	pAN52-1	gpdA	*P. griseoroseum* PG63 and T73	The ability to release P_i_ from cereals commonly used for pig feed suggest the potential application of Phytase produced by *P. griseoroseum* T73 in the animal nutrition industry.	(Ribeiro Corrêa et al. 2014)
**Manganese Peroxidase**	*Ceriporiopsis subvermispora*	MnP50297, MnP15798, MnP11743, and MnP124076	pACYCDuet-1	T7	*E. coli* BL21 (DE3)	Lignin degradation, Environmental remediation.	(Lin et al. 2017)
**Laccase**	*Coprinopsis cinerea*	Lcc9	pPIC9K	AOX1	*P. pastoris* GS115	Dye decolourisation , wastewater treatment.	(Xu et al. 2018)
**Glucose oxidase**	*Cladosporium neopsychrotolerans*	CngoxA	pPIC9	AOX1	*P. pastoris* GS115	Bread baking industry, washing detergent and aquatic feed additive industries.	(Ge et al. 2020)
**Glutaminase**	*Aspergillus oryzae*	Ao*glsA*	pKK223-3	T7	*E. coli* BL21 (DE3)	Salt-tolerant glutaminase is required for brewing high quality soy sauce with a high L-glutamic acid concentration.	(Masuoa et al. 2004)
**L-Asparaginase**	*Aspergillus terreus*	asparaginase gene	pET-28a	T7	*E. coli* BL21 (DE3)	L-asparaginase is a therapeutic agent for the treatment of a variety of lymphoproliferative disorders and lymphoma such as acute lymphoblastic leukaemia.	(Saeed et al. 2018)
**Superoxide dismutase**	*Cordyceps militaris*	cm-SOD	pET-21a	T7	*E. coli* BL21 (DE3)	Antioxidant potential, Superoxide scavanging	(Wang et al. 2005)

## 6. Conclusion and future prospects

Fungi produce myriad biocatalysts which find diverse applications in a range of industrial processes. On account of their ability to utilise low-value substrates, amenability to manipulation, and ability to generate enzymes in copious titres, they are preferred choice for production of industrial biocatalysts. Among industrial enzymes, 60% are sourced from about 25 fungal genera. Pertaining to rising number of applications, rapid growth in demand of mycozymes is indicative of their suitability in biofuel, food, detergent, pharmaceutical and nutraceutical industries. To overcome the bottle-neck of cost-effectiveness, a multi-pronged approach is required. Firstly, a more comprehensive understanding of dynamics of fungal growth and enzyme biosynthesis should be developed followed by development of state-of-the-art modules for submerged and solid-state cultures. Further, development of strains expressing robust and multifunctional (chimeric) enzymes using recombinant DNA technology, high-throughput screening of novel isolates, metagenomic screening, *in silico* enzyme engineering, site-directed mutagenesis, and directed evolution will pave a way to cater future demands. Recent advents such as CRISPR/Cas9 genome editing, number of available fungal genome sequences and knowledge of omics hold promises for development of robust fungal strains in near future.
